# Effects of Liming on Forage Availability and Nutrient Content in a Forest Impacted by Acid Rain

**DOI:** 10.1371/journal.pone.0039755

**Published:** 2012-06-28

**Authors:** Sarah E. Pabian, Nathan M. Ermer, Walter M. Tzilkowski, Margaret C. Brittingham

**Affiliations:** School of Forest Resources, Pennsylvania State University, University Park, Pennsylvania, United States of America; Roehampton University, United Kingdom

## Abstract

Acidic deposition and subsequent forest soil acidification and nutrient depletion can affect negatively the growth, health and nutrient content of vegetation, potentially limiting the availability and nutrient content of forage for white-tailed deer (*Odocoileus virginianus*) and other forest herbivores. Liming is a mitigation technique that can be used to restore forest health in acidified areas, but little is known about how it affects the growth or nutrient content of deer forage. We examined the effects of dolomitic limestone application on the growth and chemical composition of understory plants in an acidified forest in central Pennsylvania, with a focus on vegetative groups included as white-tailed deer forage. We used a Before-After-Control-Impact study design with observations 1 year before liming and up to 5 years post-liming on 2 treated and 2 untreated 100-ha sites. Before liming, forage availability and several nutrients were below levels considered optimal for white-tailed deer, and many vegetative characteristics were related to soil chemistry. We observed a positive effect of liming on forb biomass, with a 2.7 fold increase on limed sites, but no biomass response in other vegetation groups. We observed positive effects of liming on calcium and magnesium content and negative effects on aluminum and manganese content of several plant groups. Responses to liming by forbs and plant nutrients show promise for improving vegetation health and forage quality and quantity for deer.

## Introduction

Soil nutrient availability and related forage quality are known correlates of diet, health and morphometrics of many cervid species [1]–[5]. They are included in habitat suitability models of white-tailed deer (*Odocoileus virginianus*) [6] and are recommended for inclusion in deer management plans [7]. Although soil nutrients and related forage quality are acknowledged as important factors in cervid habitat quality, the current acidification and nutrient depletion in forest soils have received little attention in cervid forage quality research. Forest soil conditions in the northeastern United States, and many areas around the world, have become increasingly acidic and depleted of base cation nutrients, including calcium, magnesium, and potassium as a result of acidic deposition, forest harvesting, forest growth and maturation, and land use patterns [8]–[11]. These changes in soil conditions are affecting critical components of cervid habitat suitability, including forest vegetation health, forage availability and species composition, and nutrient content [12]–[14], yet very little is known how changes in soil conditions might affect cervids and the quality and quantity of their forage.

Strong relationships from soil nutrient availability to forage quality to white-tailed deer morphometrics have been established [4], [5], [7], but there has been no experimental evaluation of the potential for changes in soil conditions to degrade deer habitat quality. Changes in base cation nutrient availability in soils can affect abundance, species composition and nutrient content of vegetation [13], [14]. Also, poor soil conditions can result in reduced ability of vegetation to withstand high levels of browse [15]. Any changes in the calcium, magnesium, phosphorus, potassium and protein content of forage could affect deer because they are required for many life functions including bone formation and maintenance, cell function, reproduction, lactation, and antler growth [16], [17]. Particularly high levels of calcium and phosphorus are required for females during lactation and for males during antler growth [16], [18]. Also, changes in soil conditions can change phosphorus availability, which can be a limiting nutrient in ungulate herbivores [17], [19], and crude protein content of forage, a well-known correlate to deer morphometrics, can also differ among soil regions [20].

While we predicted that soil acidification could negatively affect deer through reduced forage quality and quantity, little is known about the effects of soil acidification on understory vegetation. Much of this small body of research focuses on tree saplings [14], [21] or was conducted in Europe [22]–[26]. In addition, understanding understory vegetation is important to understand future forest tree regeneration and the health of forest ecosystems [27], [28].

As a starting point to experimentally determine the effects of soil acidification and nutrient depletion on white-tailed deer, we mitigated acidic soil conditions using dolomitic limestone application and measured the response of white-tailed deer forage availability and nutrient content. Lime application is a common mitigation technique for waters and forests affected by acidic deposition [29]–[32]. Decades of liming research in Europe, and more recently the USA, have established its beneficial effects on water quality, soil nutrients, tree growth, tree health, and tree nutrient content [14], [29]–[34]. Understory plants have been studied less, and very few studies include terrestrial vertebrates [35]. Studies on the effects of liming on understory vegetation have observed increases in herbaceous and vascular plant species, changes in species composition, and changes in element concentrations in plant tissues [21], [22], [24], [26]. The aims of this study were to evaluate the current condition of deer forage in an acidified forest, and to measure the effects of liming on the chemical composition and availability of deer forage and the availability of non-forage (competing vegetation) to determine if liming can improve the forage available to white-tailed deer.

## Materials and Methods

### Study Area

This study was conducted in the Mosquito Creek watershed located in Clearfield, Cameron, and Elk counties in central Pennsylvania over the summers of 2003, 2004, and 2008. This study was part of a larger study evaluating the effects of watershed and riparian liming as a mitigation technique [36]. Our portion of the study was focused on 4, 100-ha watersheds (sites) in the Gifford Run drainage to Mosquito Creek in Clearfield county (41°11′ N, 78°17′ W).

The study area receives some of the highest levels of acidic deposition in the country [37] and once supported a world-class fishery, but stream pH and aluminum levels have become unsuitable for most fish species as a result of acidic deposition. Many characteristics of the study area indicate that the forest habitat is being impacted by the extremely acidic soil conditions, including low abundances of snails, low abundances of many common forest songbird species, reduced Ovenbird (*Seiurus aurocapilla*) clutch sizes, very little regeneration of most tree species (with the exception of *Acer rubrum*), and dominance of the understory in hay-scented fern (*Dennstaedtia punctilobula*), bracken fern (*Pteridium aquilinum*), and mountain laurel (*Kalmia latifolia*) [35], [38]. For more site details, see Pabian & Brittingham [35].

### Study Design

We conducted this study in 2003, 2004, and 2008 using a Before-After-Control-Impact (BACI) study design [39]. During year 1 (before, 2003), we collected data at all 4 sites pre-liming. During the fall and early winter between years 1 and 2, we randomly selected 2 of the 4 sites and applied approximately 4,500 kg/ha of dolomitic limestone sand. The dolomitic limestone sand contained 26% CaO and 16.4% MgO [40]. Particle size ranged from <63 µm to >3.35 mm in diameter, with about one-half of the limestone particles >2 mm and 25% of the particles between 1 and 2 mm [40]. Limestone sand was applied using a modified log skidder fitted with a lime spreader. We collected data on the limed and control sites in 2004 and again in 2008 after liming to provide the before-after and the control-impact comparisons. Within each of the 4 sites, we established 17 survey points (68 points total) where vegetation measurements took place in 2003 and 2004. In 2008, we randomly selected 8 of the 17 survey points to resample. The survey points were separated by at least 200 m, and were the same survey points used by Pabian & Brittingham [35].

### Vegetation Sampling

We used a clipped-plot method similar to Conroy et al. [41] to sample understory vegetation. We clipped all understory vegetation up to the height of 2 meters within 4, 1-m^2^ plots at survey points. We completed vegetation collection in July and August. Our plots were located in 3 different locations around points located 10 m from the center of each survey point in the directions of 30°, 120°, 210°, and 300°. The 3 plots were located above and to the right, above and to the left, and centered below the points, with a different plot sampled each year. We clipped all of the current year’s growth in each plot and grouped it in 11 categories: oak species (mostly *Quercus rubra* and *Q. alba*, with less *Q. velutina* and *Q. prinus*), red maple *(Acer rubrum.*), other tree species (mostly *Nyssa sylvatica, Sassafras albidum*, *Amelanchier spp.*, and *Hamamelis virginiana*), blueberry/huckleberry (mostly *Vaccinium* spp. and *Gaylussacia baccata*), other shrub species (mostly *Kalmia latifolia*), teaberry (*Gaultheria procumbens*), greenbrier (*Smilax* spp.), forbs, bracken fern (*Pteridium aquilinum*), other fern species (mostly *Dennstaedtia punctilobula*), and grasses. For herbaceous plants, we clipped the entire aboveground portion. For woody species, we identified the current year’s growth using bud scars and stem coloration. For species where current year’s growth was difficult to identify, we collected material from the end of the twig to 2 cm below the last leaf on primary and secondary stems. Because sampling was spread across up to 5 weeks, we stratified sampling to rotate among sites to ensure the timing of cutting would not bias our results.

The vegetation categories were based on deer forage, distribution and abundance, and sampling ease. Six categories – oak species, red maple, other tree species, Smilax species, forbs, and grasses – were classified as white-tailed deer forage, and the other 5 categories were classified as non-forage [6], [42]–[45]. Categories that contained many different species were necessary to collect sufficient vegetation for measuring changes in biomass and chemical content. All oak species were combined because of difficulty in identifying several oak species by young saplings and their uneven distribution across the study sites. Red maple, bracken fern, blueberry/huckleberry and teaberry were very abundant and kept as individual categories. All other tree species were less abundant and thus grouped together. We dried all samples in brown paper bags at 75°C until mass remained constant between days (always more than 72 hours). After drying, we weighed all samples to calculate biomass.

After drying and weighing, we ground the samples of the deer forage vegetation in a Wiley mill with a 1-mm mesh screen, and analyzed them for nutrient content. We analyzed individually the samples collected at each survey point for oak species, red maple, and forbs. We pooled samples collected across each site for other tree species, greenbrier, and grass because we did not have enough vegetation to analyze by point. Two representative samples were drawn from the pooled samples for analysis. We did not chemically analyze the other vegetation groups because they were not considered forage of white-tailed deer. We analyzed the 2003 and 2004 vegetation for calcium, magnesium, potassium, phosphorus, and crude protein. We analyzed the 2008 vegetation for calcium, magnesium, potassium, phosphorus, aluminum and manganese. We included aluminum and manganese in 2008 because liming was predicted to result in decreases in the availability of aluminum and manganese to plants (both increase in availability as soil become acidified) [46]. We were unable to include crude protein in the analyses in 2008. Chemical analyses were conducted at the Pennsylvania State University Agricultural Analytical Services Lab or at Agri-Analysis, Inc., Leona, Pennsylvania using acid digestion [47]. Ammonium concentration was determined colorimetrically using extra-alkaline Nessler reagent, and was converted to crude protein equivalents by multiplying by a factor of 6.25.

### Soil Sampling

We used the Oa-horizon soil samples collected by Rummel [48] to correlate with the vegetation variables measured in the first year of this study. Soils were collected at each survey point and analyzed at the Pennsylvania State University Agricultural Analytical Services Lab for pH, exchangeable calcium, magnesium, potassium, and phosphorus [49], [50]. The results of the effects of liming on soil calcium and pH were reported in Pabian et al. [38] with positive effects of liming on soil pH (increased from 3.83 to 4.69 on limed sites), calcium (increased from 5.31 to 13.30 cmol/kg on limed sites) and magnesium (increased from 1.52 to 8.28 cmol/kg on limed sites) and no effects on potassium or phosphorus.

### Statistical Analysis

All statistical analyses were completed in R (Version 2.12.0, www.r-project.org). We used the *glm* function to perform generalized linear models relating initial (before liming) understory vegetation availability and nutrient content to soil conditions. We assessed the relationship between vegetation and soil variables using 95% confidence intervals to indicate magnitude and uncertainty in slope estimates. We considered slope estimates with confidence intervals that excluded zero to indicate significant relationships between the soil measure and vegetation variable. We modeled biomass for each vegetation group, for total deer-forage vegetation, for total non-forage vegetation or for the proportion of total vegetation that was forage as the response variable and Oa-horizon soil pH, calcium, magnesium, potassium, or phosphorus as the predictor variable. We modeled the calcium, magnesium, potassium, phosphorus, and crude protein content of the vegetation groups with point-level data (oaks, red maple and forbs) as response variables, with soil variables as predictor variables. We also modeled biomass of deer-forage vegetation as the response variables and biomass of non-forage vegetation as the predictor variables to examine the potential for competition. All models had a Gaussian error structure and biomass and nutrient-content variables were log transformed for normality.

To evaluate the effects of liming, we used mixed-effects models to conduct repeated measures analyses. We included only the data collected at the same eight points in each site. For all biomass variables and for the nutrient content variables of oaks, red maple, and forbs, the models included fixed treatment (limed or control) and time (years 2003, 2004 and 2008) effects, fixed treatment by time interaction effect, random site (four sites) effect, and random point within site (eight points within each of the four sites) effect. For the nutrient content variables of other preferred deer browse categories, the model included the same fixed- and random-effects, except the random point within site was changed to random sample from within site because we took two samples from the combined point samples for chemical analyses. The random terms in the model structure the error to allow the use of point-level, repeated measures data without committing pseudoreplication [51]. To test for the effect of liming, we used the time by treatment interaction term. By using the time by treatment interaction, we examined the difference in how the control and treatment sites changed between before liming to after liming, as in BACI analysis [39]. We used the *lmer* function from the *lme4* package in R to analyze the data [52]. We log transformed vegetation nutrient content variables, and any non-normally distributed biomass variables.

We assessed the uncertainty in the time by treatment interaction parameter estimates using 95% confidence limits by generating Markov Chain Monte Carlo (MCMC) samples from the posterior distribution of each parameter estimate using the *mcmcsamp* function in the R package *lme4* [52] and computing the Bayesian highest posterior density (HPD) 95% confidence limits of the MCMC samples using the *HPDinterval* function in the R package *coda* [53]. We considered confidence limits that excluded zero to indicate a time by treatment interaction and an effect of liming on the variable measured. We reported interaction effect parameter estimates with their confidence intervals (CI) and the changes that occurred on control and treatment sites with their standard errors to indicate the direction and magnitude of the liming effect.

We only collected data on understory vegetation aluminum and manganese content in 2008, therefore, we could not analyze these data as a BACI experiment. We used the *lmer* function from the *lme4* package in R to analyze these data [52]. We included lime as a fixed effect and site as a random effect because we had repeated samples taken from within sites (our experimental units). We used the parameter estimates for the treatment factor and confidence limits as calculated above using MCMC and HPD intervals.

## Results

### Initial Conditions

**Figure 1:**
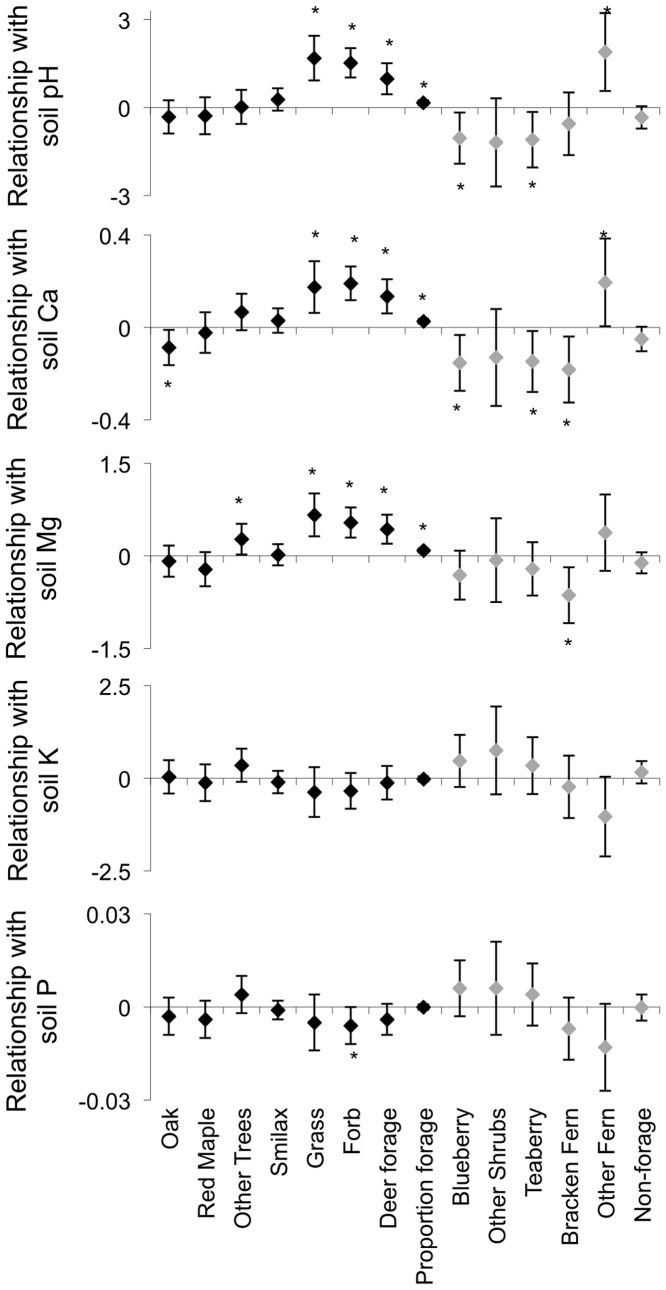
Slopes of relationships between soil and vegetation biomass. Measures include soil chemical parameters (pH, calcium, magnesium, potassium, and phosphorus in cmol/kg) and vegetation biomass (kg/ha) of deer forage (black diamonds) and non-forage (gray diamonds) with 95% confidence intervals measured in central Pennsylvania, USA, 2003. *Confidence intervals that exclude zero indicate a significant relationship.

**Figure 2:**
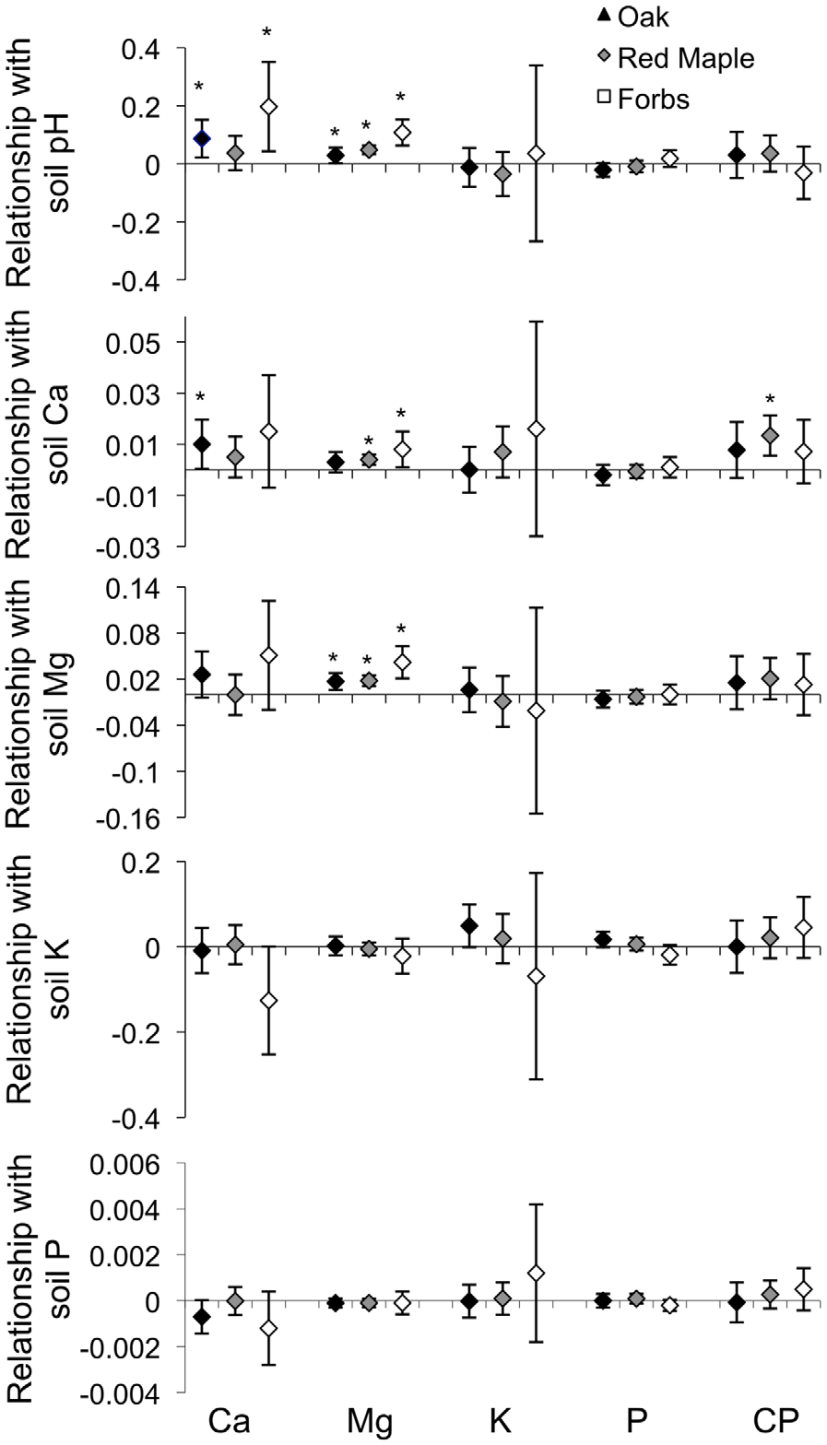
Slopes of the relationships between soil and vegetation nutrients. Measures include soil chemical parameters (pH, calcium, magnesium, potassium, and phosphorus in cmol/kg) and vegetation nutrient and crude protein (CP) content (percent dry weight) of oaks, maples and forbs with 95% confidence intervals in Pennsylvania, USA, 2003. *Confidence intervals that exclude zero indicate a significant relationship.

Before liming, the biomass and chemical composition of vegetation were variable among plant categories and many were related to soil condition (Table 1, Figs. 1 and 2). As a group, preferred forage vegetation biomass was positively related to soil pH, calcium and magnesium (Fig. 1). Specifically, both grass and forb biomass were positively related to soil pH, calcium and magnesium; other tree species biomass was positively related to soil magnesium; and oak biomass was negatively related to soil calcium (Fig. 1). Forb biomass was also negatively related to soil phosphorus (Fig. 1). The proportion of understory vegetation that was considered deer forage was also positively related to soil pH, calcium and magnesium (Fig. 1). As a group, non-forage vegetation was unrelated to soil conditions, although blueberry/huckleberry biomass was negatively related to soil pH and calcium; teaberry biomass was negatively related to soil pH and calcium; bracken fern biomass was negatively related to soil calcium and magnesium; and other fern species biomass was positively related to soil pH and calcium (Fig. 1).

**Table 1:** Mean biomass (kg/ha) and chemical composition (percent dry weight of calcium, magnesium, potassium, phosphorus, and crude protein) of deer forage and non-forage vegetation with standard errors collected at four study sites in 2003 in Pennsylvania, USA.

Category	Biomass		Ca		Mg		K		P		CP	
**Forage**												
Oak	13.59	(2.62)	0.58	(0.02)	0.17	(0.01)	0.72	(0.01)	0.11	(0.01)	10.36	(0.26)
Red Maple	19.55	(6.00)	0.63	(0.02)	0.13	(0.01)	0.58	(0.02)	0.12	(0.01)	7.71	(0.21)
Other trees	18.56	(5.32)	0.90	(0.11)	0.21	(0.01)	1.04	(0.03)	0.16	(0.01)	10.20	(0.32)
Forbs	11.87	(2.34)	0.74	(0.04)	0.30	(0.02)	2.07	(0.11)	0.15	(0.01)	11.35	(0.31)
Grass	22.90	(6.94)	0.19	(0.03)	0.10	(0.02)	1.64	(0.14)	0.16	(0.02)	9.00	(0.21)
Smilax	1.44	(0.64)	0.58	(0.04)	0.13	(0.01)	1.26	(0.10)	0.11	(0.01)	9.93	(0.50)
Total forage	87.90	(12.84)	–	–	–	–	–	–	–	–	–	–
**Non-forage** *												
Blueberry	136.36	(19.56)	–	–	–	–	–	–	–	–	–	–
Other shrubs	118.54	(48.33)	–	–	–	–	–	–	–	–	–	–
Teaberry	28.32	(8.11)	–	–	–	–	–	–	–	–	–	–
Bracken fern	211.40	(32.18)	–	–	–	–	–	–	–	–	–	–
Other fern	182.17	(31.69)	–	–	–	–	–	–	–	–	–	–
Total non-forage	676.79	(73.30)	–	–	–	–	–	–	–	–	–	–

* We did not chemically analyze non-forage vegetation.

For nutrient content, oak calcium content was positively related to soil pH and calcium, and oak magnesium content was positively related to soil pH and magnesium (Fig. 2). Red maple magnesium content was positively related to soil pH, calcium and magnesium and red maple crude protein content was positively related to soil calcium (Fig. 2). Forb calcium content was positively related to soil pH, and forb magnesium content was positively related to soil pH, calcium and magnesium (Fig. 2). We detected no other strong relationships among the remaining vegetation-soil pairs.

We also observed a negative relationship between the biomass of deer forage and non-forage vegetation (slope (95% CI): −0.043 (−0.751, −0.098)).

### Effects of Liming

**Figure 3:**
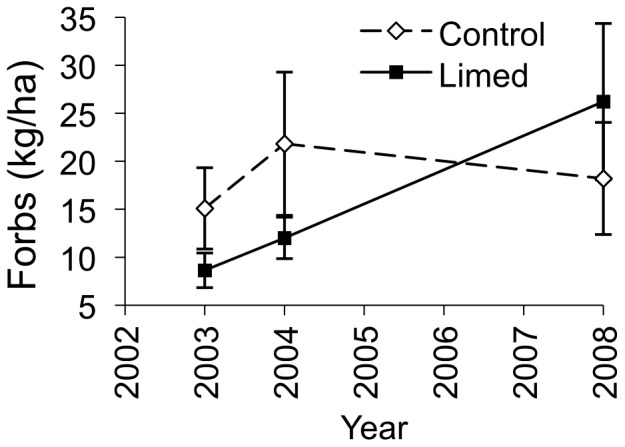
Biomass of forbs. Forb biomass (± standard error) on control and lime-treated sites before (2003) and after (2004, 2008) limestone sand application in Pennsylvania, USA. *Confidence interval of the time by treatment parameter estimate excludes zero, indicating an effect of liming.

We observed a positive effect of liming on forb biomass (Fig. 3). Forb biomass remained similar on control sites from before liming to five years after liming (3.11±7.22 change kg/ha), while it increased on limed sites (15.94±7.98 change kg/ha). We found no substantial effect of liming on the other understory vegetation biomass variables (Table S1).

**Figure 4:**
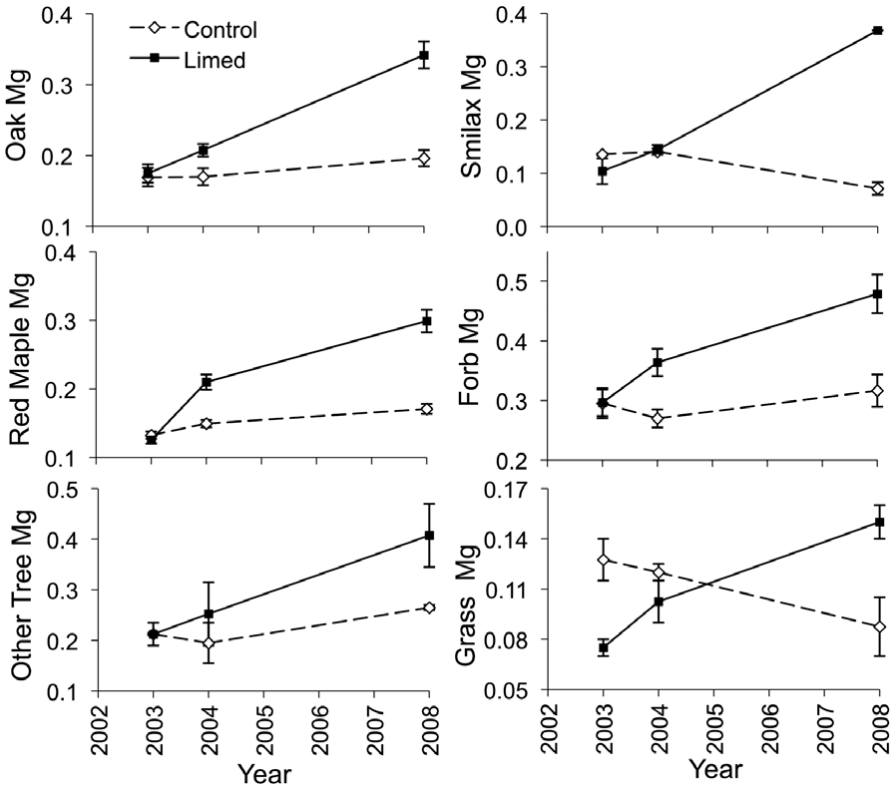
Magnesium content of forage. Magnesium content (percent dry weight ± standard error) of oak species, red maple, other tree species, *Smilax* species, forbs, and grasses on control and lime-treated sites before (2003) and after (2004, 2008) limestone sand application in Pennsylvania, USA. *Confidence interval of the time by treatment parameter estimate excludes zero, indicating an effect of liming.

**Figure 5:**
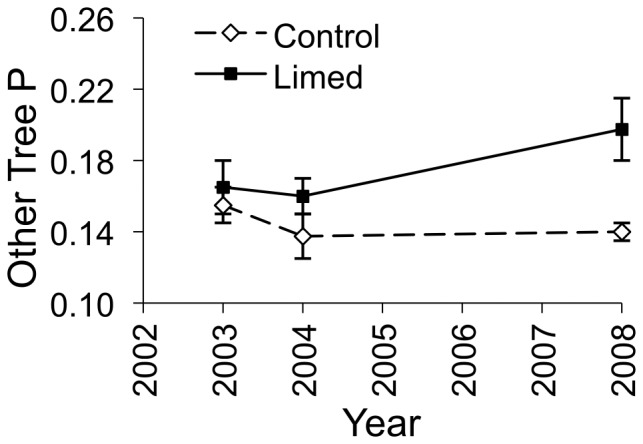
Calcium content of forage. Calcium content (percent dry weight ± standard error) of *Smilax* species and grasses on control and lime-treated sites before (2003) and after (2004, 2008) limestone sand application in Pennsylvania, USA. *Confidence interval of the time by treatment parameter estimate excludes zero, indicating an effect of liming.

**Figure 6:**
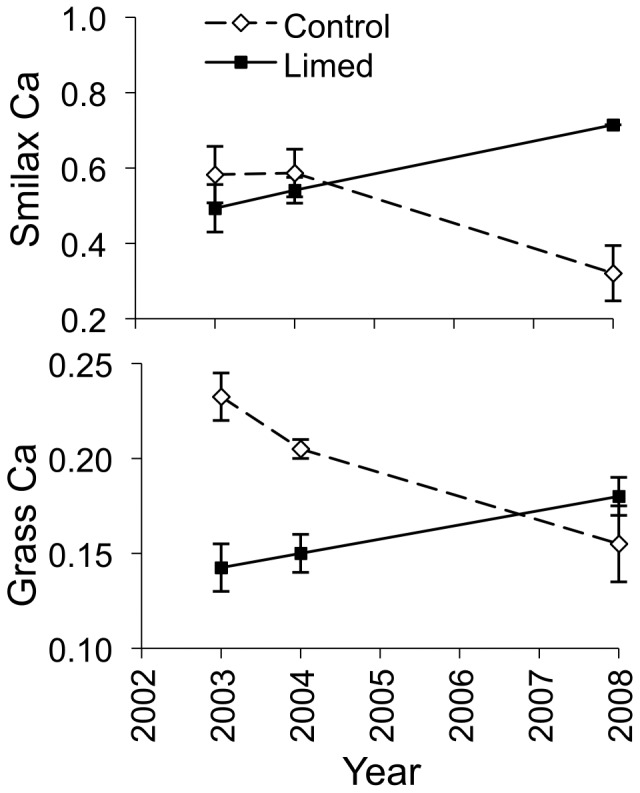
Phosphorus content of forage. Phosphorus content (percent dry weight ± standard error) of other trees species (excluding oaks and red maple) on control and lime-treated sites before (2003) and after (2004, 2008) limestone sand application in Pennsylvania, USA. *Confidence interval of the time by treatment parameter estimate excludes zero, indicating an effect of liming.

We observed effects of liming on many of the chemical characteristics of plants. We observed positive effects of liming on oak species magnesium content, red maple magnesium content, forb magnesium content, other tree species phosphorus and magnesium content, grass calcium and magnesium content, and greenbrier calcium and magnesium content (Figs. 4, 5, 6; Table S2). Liming did not affect the potassium or protein content of any plant (Table S2).

We observed several differences between aluminum and manganese content in the vegetation from control and limed sites five years after liming. Control site vegetation had higher concentrations of manganese than limed sites in oak species and red maple (Table 2). Grass from control sites had higher concentrations of aluminum than limed sites (Table 2).

**Table 2:** Concentration (ppm) of manganese (Mn) and aluminum (Al) with standard errors in groups of deer forage sampled in control and lime-treated sites in 2008 (five years after lime application) in Pennsylvania, USA, and the treatment parameter estimates with confidence intervals (CI).

Category	Control		Limed		Treatment (CI)	
Oak Mn	2906.3	(350.9)	1642.6	(300.1)	−0.58	(−1.06, −0.12) *
Oak Al	21.1	(3.5)	20.0	(2.6)	−0.21	(−0.83, 0.28)
Red Maple Mn	2643.2	(174.1)	1278.2	(365.6)	−0.88	(−1.57, −0.31) *
Red Maple Al	9.7	(1.1)	8.3	(0.9)	−0.15	(−0.58, 0.27)
Other Trees Mn	1920.3	(188.4)	1428.4	(318.9)	−0.31	(−0.69, 0.11)
Other Trees Al	88.8	(55.8)	23.5	(1.8)	−0.99	(−2.21, 0.22)
Forbs Mn	1304.5	(19.5)	879.6	(10.6)	−0.52	(−1.05, 0.01)
Forbs Al	143.6	(100.8)	54.6	(27.8)	−0.43	(−2.19, 0.97)
Grass Mn	540.6	(143.8)	449.8	(53.3)	−0.15	(−0.57, 0.38)
Grass Al	22.4	(3.3)	15.3	(1.4)	−0.38	(−0.73, −0.02) *
Smilax Mn	211.2	(116.8)	660.8	(196.3)	1.28	(−0.09, 2.64)
Smilax Al	5.3	(2.1)	24.2	(13.0)	1.48	(−0.03, 2.93)

* Confidence intervals exclude zero, indicating a difference between control and lime-treated sites.

## Discussion

Deer forage availability and nutrient content were both related to and affected by soil conditions in this study. While we did not study how soil conditions affect white-tailed deer, we experimentally established a causal pathway for the quality of deer forage to be affected by changes in soil acidity and nutrient availability, and other studies have observed relationships between both soil and forage quality and deer health and morphometrics [4], [5], [7], [20].

### Initial Conditions

Results from before liming indicate that forage biomass and nutrient content are of moderate to low quality for white-tailed deer, although comparable biomass and nutrient content information was difficult to find. Also, the relationships between soil pH, calcium, and magnesium and the biomass and nutrient content of deer forage indicates a potential for soil acidification to negatively affect deer forage availability and quality, however, these were correlative results and only indicate a potential causative link.

We observed low deer forage biomass compared to other studies [54], [55], with only approximately 11% of the available understory vegetation biomass in deer forage. Vegetation types not typically used as forage by deer dominated the understory vegetation, including mountain laurel (*Kalmia latifolia*), blueberry (*Vaccinium spp.*), and fern. These vegetation types are known to have associations with acidic soils and can outcompete forage vegetation and reduce tree regeneration [56]–[59].

In general, crude protein content of deer forage exceeded minimum requirements but were not considered “optimum” by many studies. None of the forage categories we measured had levels optimal for post weaning fawns, antlerogenesis, or lactating females [16], [60]–[63]. The forage calcium levels we measured were sufficient, but not optimal when compared to other studies [16], [63]–[64]. The deer forage calcium levels observed in this study were higher than the levels reported necessary for fawns and deer in antlerogenisis by other researchers (greater than 0.60%) [16], [63], with the exception of grasses. However, Smith et al. [64] observed much higher calcium levels in deer forage in an area with a healthy deer herd (larger body sizes and larger populations), and comparable calcium levels in an area with an unhealthy deer herd when compared to our observation. Phosphorus content of forage was lower than what has been reported necessary for post-weaning fawn maintenance and growth (>0.25%), adult winter maintenance (0.30%), and antler development (0.56%) [16], [63]. However, Grasman & Hellgren [65] suggest 0.14% is sufficient. There is very little information available on the amount of magnesium in deer forage or the amount of magnesium that is needed in deer diets, but magnesium is needed for bone formation and antler growth [16].

### Effects of Liming

Liming successfully increased soil pH, calcium and magnesium on our study sites [38]. As a result, liming also had a positive effect on the calcium, magnesium and phosphorus content of deer forage and the availability of forbs, all of which could benefit white-tailed deer forage quality. Dulière et al. [24] also observed rapid responses of the forb layer to dolomitic lime additions. Forbs are an important food item for white-tailed deer, especially in spring and early summer months, when forbs can compose over 75% of deer diets [66], [67]. Forb availability may also be critical for early spring survival and fawn growth because forbs are some of the first new growth in the spring, are easy to digest, and contain high levels of nutrients, including calcium and protein [68]. The 2.7 fold increase in forb availability observed in this study could represent a substantial increase in forage availability for herbivores. Also, forbs had some of the highest nutrient levels of crude protein, calcium, magnesium, potassium, and phosphorus compared to the other vegetation groups and may serve as an important source for nutrients.

Increases in calcium and magnesium content of browse could benefit deer because they require large amounts of calcium and lesser amounts of magnesium for antler growth [16] and large amounts of calcium for lactation [18]. Healthier vegetation with higher nutrient availability can better withstand high levels of browse [15]. Also, forage magnesium levels can play a large role in the distribution of large ungulate herbivores [69]. While we observed positive responses in magnesium content for all of our plant groups, we only observed significant increases in calcium content in grasses and greenbrier, although all plant groups showed strong positive trends. This response of calcium content to liming may reflect the smaller increase in soil calcium relative to the increase in magnesium observed after liming. After liming, we found a 5.5 fold increase in soil magnesium, while we only found a 4.1 fold increase in soil calcium even though the limestone contained more calcium than magnesium [38], [40]. Other studies found similar results of observing greater increases in magnesium plant content then calcium after dolomitic lime application [21], [33], [70].

Also, in areas with low soil nutrient availability (like at our study sites), forage mineral content can become a more important factor in diet selection than protein and energy, to the point where vegetation selected may have lower levels of protein and energy than other available vegetation [71]. Jones et al. [7] observed that in areas with generally poor soil fertility, deer were larger in areas with higher calcium availability, even as these sites had less vegetative protein availability. These results indicate the importance of nutrients, like calcium and magnesium, in the diets of cervids.

The forage group containing tree saplings other than oaks and red maple showed a positive response in phosphorus content to liming and several other forage groups also showed positive trends. Long et al. [33] also found a trend toward higher phosphorus content in sugar maple foliage after dolomitic lime application. After liming, phosphorus could potentially increase in availability resulting from desorption from aluminum compounds or increased mineralization, or it could decrease by precipitation of calcium phosphates [19]. Increases in vegetation phosphorus could benefit white-tailed deer, because most of the understory vegetation at our study sites contained less phosphorus than was reported necessary for post-weaning fawns, winter deer forage, and antler growing [16], [63].

While we observed many positive effects of liming on understory plant nutrient levels and forb biomass, we observed no significant effect on the vegetation biomass in other categories. The amount and type of understory vegetation within plots varied widely and potentially our sample size was not large enough to detect treatment effects within that variability. In fact, we detected a pattern that the biomass of deer forage tended to respond in a positive direction to liming while non-forage vegetation tended to respond in a negative direction. However, in another measure of understory vegetation used to examine bird habitat [38], we only observed an effect of liming on percent forb cover, which agreed with this study. Potentially, understory vegetation growth was limited by sunlight, competition with fern, or other nutrient limitations, as many researchers have found the highest growth response to liming when combined with fertilizer, fern reduction, or canopy thinning [72], [73]. Alternatively, the improved nutrient content of the vegetation on limed plots could have resulted in increased deer forage if deer preferentially feed on more nutritious vegetation.

As predicted, we also observed lower concentrations of aluminum and manganese in the vegetation on limed sites, which can be toxic to plants and can affect nutrient balance, and photosynthesis [74]–[76]. Previous liming studies have also documented similar decreases of aluminum and manganese in vegetation [21], [26], [34]. Reductions in plant aluminum and manganese content could represent healthier vegetation that can withstand more browse. Also, high levels of aluminum can be toxic to wildlife and have been linked to reproductive problems in birds [77], [78].

### Conclusions

Liming had positive effects on understory forb biomass and vegetation chemistry, with increases in calcium, magnesium, and phosphorus contents and decreases in the metals aluminum and manganese five years post-liming. These changes in vegetation represent improved habitat quality for white-tailed deer. This study was designed to evaluate a method of liming that could be used easily by a land managers to mitigate the effects of acidic deposition with several goals (improve water quality, forest health, wildlife health) and our results show promise for the technique to improve vegetation quality for deer forage. A longer-term study may be required to determine if liming is improving understory vegetation composition and tree regeneration, further improving habitat composition for deer. The next step should be to monitor deer population and health responses to experimental lime application.

## Supporting Information

Table S1 Dry mass (kg/ha) of the current year’s growth of each understory plant group, separated by deer forage and non-forage for white-tailed deer, and the proportion of total vegetation classified as forage on control and lime-treated sites before (2003) and after (2004, 2008) lime application with standard errors, and the estimates of the time by treatment interaction term with confidence intervals (CI).
(PDF)

Table S2 Percent dry weight (SE) of calcium (Ca), magnesium (Mg), potassium (K), phosphorus (P), and crude protein (CP) in the six categories of deer forage sampled in control and lime-treated sites before (2003) and after (2004, 2008) lime application, and the estimates of the time by treatment interaction term with confidence intervals (CI).
(PDF)

## References

[1] Herfindal I, Sæther B-E, Solber E J, Andersen R, Høgda KA (2006). Population characteristics predict responses in moose body mass to temporal variation in the environment.. Journal of Animal Ecology.

[2] Gaillard J-M, Delorme D, Boutin J-M, Van Laere G, Boisaubert B (1996). Body mass of roe deer fawns during winter in 2 contrasting populations.. Journal of Wildlife Management.

[3] Lehoczki R, Erdélyi K, Sonkoly K, Szemethy L, Csányi S (2011). Iodine distribution in the environment as a limiting factor for roe deer antler development.. Biological Trace Element Research.

[4] Gill J (1956). Regional differences in size and productivity of deer in West Virginia.. Journal of Wildlife Management.

[5] Strickland BK, Demarais S (2000). Age and regional differences in antlers and mass of white-tailed deer.. Journal of Wildlife Management.

[6] Crawford HS, Marchinton RL (1989). A habitat suitability index for white-tailed deer in the Piedmont.. Southern Journal of Applied Forestry.

[7] Jones PD, Strickland BK, Demarais S, Rude BJ, Edwards SL (2010). Soils and forage quality as predictors of white-tailed deer *Odocoileus virginianus* morphometrics.. Wildlife Biology.

[8] Andersson F (1986). Acidic deposition and its effects on the forests of Nordic Europe.. Water, Air and Soil Pollution.

[9] Adams MB, Burger JA, Jenkins AB, Zelanzy L (2000). Impact of harvesting and atmospheric pollution on nutrient depletion of eastern US hardwood forests.. Forest Ecology and Management.

[10] Driscoll CT, Lawrence GB, Bulger AJ, Butler TJ, Cronan CS (2001). Acidic deposition in the Northeastern United States: Sources and inputs, ecosystem effects, and management strategies.. BioScience.

[11] Hamburg SP, Yanai RD, Arthur MA, Blum JD, Siccama TG (2003). Biotic control of calcium cycling in northern hardwood forests: Acid rain and aging forests.. Ecosystems.

[12] Horsley SB, Long RP, Bailey SW, Hallett RA, Hall TJ (2000). Factors associated with the decline disease of sugar maple on the Allegheny Plateau.. Canadian Journal of Forest Research.

[13] Demchik MC, Sharpe WE (2001). Forest floor plant response to lime and fertilizer before and after partial cutting of a northern red oak stand on an extremely acidic soil in Pennsylvania, USA.. Forest Ecology and Management.

[14] Juice SM, Fahey TJ, Siccama TG, Driscoll CT, Denny EG (2006). Response of sugar maple to calcium addition to northern hardwood forest.. Ecology.

[15] Maschinski J, Whitham TG (1989). The continuum of plant responses to herbivory: the influence of plant associations, nutrient availability, and timing.. American Naturalist.

[16] French CE, McEwen LC, Magruder ND, Ingram RH, Swift RW (1956). Nutrient requirements for growth and antler development in the white-tailed deer.. Journal of Wildlife Management.

[17] McDowell LR (1985). Nutrition of grazing ruminants in warm climates.. Academic Press, Inc., Orlando, FL, USA.

[18] Simkiss K (1967). Calcium in reproductive physiology.. New York: Chapman and Hall, London and New York.

[19] Haynes RJ (1982). Effects of liming on phosphate availability in acid soils.. Plant and soil.

[20] Jones PD, Demarais S, Strickland BK, Edwards SL (2008). Soil region effects on white-tailed deer forage protein content.. Southeastern Naturalist.

[21] Ljungström M, Nihlgård B (1995). Effects of lime and phosphate additions on nutrient status and growth of beech (*Fagus sylvatica* L.) seedlings.. Forest Ecology and Management.

[22] Rodenkirchen H (1992). Effects of acidic precipitation, fertilization and liming on the ground vegetation in coniferous forests of southern Germany.. Water, Air, and Soil Pollution.

[23] Falkengren-Grerup U, Quists ME, Tyler G (1995). Relative importance of exchangeable and soil solution cation concentrations to the distribution of vascular plants.. Environmental and Experimental Botany.

[24] Dulière JF, Carnol M, Dalem S, Remacle J, Malaisse F (1999). Impact of dolomite lime on the ground vegetation and on potential net N transformations in Norway spruce (*Picea albies* (L.) Karst.) and sessile oak (*Quercus petraea* (Matt.) Lieb.) stands in the Belgian Ardenne.. Annals of Forest Science.

[25] Olsson BA, Kellner O (2002). Effects of soil acidification and liming on ground flora establishment after clear-felling of Norway spruce in Sweden.. Forest Ecology and Management.

[26] Grønflaten LK, Amundsen L, Frank J, Steinnes E (2005). Influence of liming and vitality fertilization on trace element concentrations in Scots pine forest soil and plants.. Forest Ecology and Management.

[27] Baker TT, Van Lear DH (1998). Relations between density of rhododendron thickets and diversity of riparian forests.. Forest Ecology and Management.

[28] Gilliam FS (2007). The ecological significance of the herbaceous layer in temperate forest ecosystems.. BioScience.

[29] Huettl RF, Zoettl HW (1993). Liming as a mitigation tool in Germany’s declining forests–reviewing results from former and recent trials.. Forest Ecology and Management.

[30] Gunn J, Sein R, Keller B, Beckett P (2001). Liming of acid and metal contaminated catchments for the improvement of drainage water quality.. Water, Air, and Soil Pollution.

[31] Traaen TS, Frogner T, Hindar A, Kleiven E, Lande A (1997). Whole-catchment liming at Tjønnstrond, Norway: An 11-year record..

[32] Hindar A, Wright RF, Nilsen P, Larssen T, Høgberget R (2003). Effects of stream water chemistry and forest vitality after whole-catchment application of dolomite to a forest ecosystem in southern Norway.. Forest Ecology and Management.

[33] Long RP, Horsley SB, Lilja PR (1997). Impacts of forest liming on growth and crown vigor of sugar maple and associated hardwoods.. Canadian Journal of Forest Research.

[34] Misson L, Ponette Q, André F (2001). Regional scale effects of base cation fertilization on Norway spruce and European beech stands situated on acid brown soils: soil and foliar chemistry.. Annals of Forest Science.

[35] Pabian SE, Brittingham MC (2007). Terrestrial liming benefits birds in an acidified forest in the Northeast.. Ecological Applications.

[36] Sharpe WE, Brittingham MC, Tzilkowski WM, Swistock BR, Bohnenblust AK (2006). Evaluation of whole watershed and riparian wetland liming to mitigate acidity, Final Report to The Pennsylvania Department of Environmental Protection.. Grant NO.

[37] National Atmospheric Deposition Program NSRP-3 (2010). NADP Program Office, Illinois State Water Survey, Champaign, Illinois.. http://nadp.sws.uiuc.edu.

[38] Pabian SE, Rummel SM, Sharpe WE, Brittingham MC (2012). Terrestrial liming as a restoration technique for acidified forest ecosystems.. International Journal of Forestry Research.

[39] McDonald TL, Erickson WP, McDonald LL (2000). Analysis of count data from before-after control-impact studies.. Journal of Agricultural, Biological, and Environmental Statistics.

[40] Mizel NL (2005). The transport and fate of applied limestone sand and palletized lime following timber harvest.. Thesis, Pennsylvania State University, PA, USA.

[41] Conroy MJ, Oderwald RG, Sharik TL (1982). Forage production and nutrient concentration in thinned loblolly pine plantations.. Journal of Wildlife Management.

[42] Webb WL (1959). Summer browse preferences of Adirondack white-tailed deer.. Journal of Wildlife Management.

[43] Healy WM (1971). Forage preferences of tame deer in northwest Pennsylvania clear-cutting.. Journal of Wildlife Management.

[44] Meyer MW, Brown RD, Graham MW (1984). Protein and energy content of white-tailed deer diets in the Texas Coastal Bend.. Journal of Wildlife Management.

[45] Horsley SB, Stoute SL, deCalesta DS (2003). White-tailed deer impact on the vegetation dynamics of a northern hardwood forest.. Ecological Applications.

[46] Wolt JD, Lucier AA, Haines SG (1990). Effects of acidic deposition on the chemical form and bioavailability of soil aluminum and manganese..

[47] Huang CYL, Schulte EE (1985). Digestion of plant tissue for analysis by ICP emission spectroscopy.. Communications in Soil Science and Plant Analysis.

[48] Rummel SM (2006). Short-term effects of forest liming on soil chemistry and terrestrial macroinvertebrates.. Thesis, Pennsylvania State University, PA, USA.

[49] Eckert D, Thomas Sims J, Thomas Sims J, Wolf A (1995). Recommended soil pH and lime requirement tests..

[50] Wolf AM, Beegle DB, Thomas Sims J, Wolf A (1995). Recommended soil tests for macronutrients: phosphorus, potassium, calcium, and magnesium..

[51] Pinheiro JC, Bates DM (2000). Mixed-Effects Models in S and S-Plus.. Springer, New York, NY.

[52] Bates D, Maechler M (2009). lme4: Linear mixed-effects models using S4 classes.. R package version 0.999375-31.

[53] Plummer M, Best N, Cowles K, Vines K (2009). coda: Output analysis and diagnostics for MCMC.. R package version 0.13-4.

[54] Blair RM, Short HL, Epps EA (1977). Seasonal nutrient yield and digestibility of deer forage from a young pine plantation.. Journal of Wildlife Management.

[55] Wallmo OC, Carpenter LH, Regelin WL, Gill RB, Baker DL (1977). Evaluation of deer habitat on a nutritional basis.. Journal of Range Management.

[56] Wherry ET (1920). Observations on the soil acidity of Ericaceae and associated plants in the middle Atlantic states.. Proceedings of the Academy of Natural Sciences of Philadelphia.

[57] Lyon J, Sharpe WE (1996). Hay-scented fern (*Dennstaedtia punctilobula* (Michx.) Moore) interference with growth of northern red oak (*Quercus rubra* L.) seedlings.. Tree Physiology.

[58] Sharpe WE, Halofsky JE, Yaussey DA, Hix DM, Long RP, Goebel PC (2004). Hay-scented fern (*Dennstaedtia punctilobula*) and sugar maple (*Acer saccharum*) seedling occurrence with varying soil acidity in Pennsylvania..

[59] Royo AA, Carson WP (2006). On the formation of dense understory layers in forests worldwide: consequences and implications for forest dynamics, biodiversity, and succession.. Canadian Journal of Forest Research.

[60] Murphy DA, Coates JA (1966). Effects of dietary protein on deer.. Transactions of the North American Wildlife and Natural Resources Conference.

[61] Ullrey DE, Youatt WG, Johnson HE, Fay LD, Bradley BL (1967). Protein requirement of white-tailed deer fawns.. Journal of Wildlife Management.

[62] Smith SH, Holter JB, Hayes HH, Silver H (1975). Protein requirement of white-tailed deer fawns.. Journal of Wildlife Management.

[63] McEwen LC, French CE, Magruder ND, Swift RW, Ingram RH (1957). Nutrient requirements of the white-tailed deer.. Transactions of the North American Wildlife Conference.

[64] Smith FH, Beeson KC, Price WE (1956). Chemical composition of herbage browsed by deer in two wildlife management areas.. Journal of Wildlife Management.

[65] Grasman BT, Hellgren EC (1993). Phosphorus nutrition in white-tailed deer: Nutrient balance, physiological responses, and antler growth.. Ecology.

[66] Crawford JS (1982). Seasonal food selection and digestibility by tame white-tailed deer in central Maine.. Journal of Wildlife Management.

[67] McCullough DR (1985). Variables influencing food habits of white-tailed deer on the George Reserve.. Journal of Mammalogy.

[68] Vangilder LD, Torgerson O, Porath WR (1982). Factors influencing diet selection by white-tailed deer.. Journal of Wildlife Management.

[69] McNaughton SJ (1988). Mineral nutrition and spatial concentrations of African ungulates.. Nature.

[70] Demchik MC, Sharpe WE (2000). Soil amendment effects on growth and sprouting success in a Pennsylvania northern red oak stand on extremely acidic soils.. Journal of Sustainable Forestry.

[71] Belovsky GE, Jordan PA (1981). Sodium dynamics and adaptations of a moose population.. Journal of Mammalogy.

[72] Schreffler AM, Sharpe WE (2003). Effects of lime, fertilizer, and herbicide on forest soil and soil solution chemistry, hardwood regeneration, and hardwood growth following shelterwood harvest.. Forest Ecology and Management.

[73] Sharpe WE, Voorhees CR, Buckley DS, Clatterbuck WK (2006). Effects of lime, fertilizer, and herbicide on herbaceous species diversity and abundance following red oak shelterwood harvest..

[74] Andersson M (1988). Toxicity and tolerance of aluminum in vascular plants.. Water, Air, & Soil Pollution.

[75] Roy AK, Sharma A, Talukder G (1988). Some aspects of aluminum toxicity in plants.. Botanical Review.

[76] St. Clair SB, Lynch JP (2005). Element accumulation patterns of deciduous and evergreen tree seedlings on acid soils: implications for sensitivity to manganese toxicity.. Tree Physiology.

[77] Scheuhammer AM (1987). The chronic toxicity of aluminum, cadmium, mercury, and lead in birds: A review.. Environmental Pollution.

[78] Nyholm NEI (1981). Evidence of involvement of aluminum in causation of defective formation of eggshells and impaired breeding in wild passerine birds.. Environmental Research.

