# Supplementary material for: Effects of Liming on Forage Availability and Nutrient Content in a Forest Impacted by Acid Rain
Source: PLoS One. 2012 Jun 28;7(6):e39755. doi: 10.1371/journal.pone.0039755 (PMC3386234; doi:10.1371/journal.pone.0039755)
Supplement: Table S1 — Dry mass (kg/ha) of the current year’s growth of each understory plant group, separated by deer forage and non-forage for white-tailed deer, and the proportion of total vegetation classified as forage on control and lime-treated sites before (2003) and after (2004, 2008) lime application with standard errors, and the estimates of the time by treatment interaction term with confidence intervals (CI). (PDF) [file pone.0039755.s001.pdf]

Table S1. Dry mass (kg/ha) of the current year's growth of each understory plant group, separated by deer forage and non-forage for white-tailed deer, and the proportion of total vegetation classified as forage on control and lime-treated sites before (2003) and after (2004, 2008) lime application with standard errors, and the estimates of the time by treatment interaction term with confidence intervals (CI).

	Control			Limed				
Plant group	2003	2004	2008	2003	2004	2008	Interaction (CI)	
Oak	8.58 (1.87)	9.85 (1.85)	11.73 (3.06)	18.59 (4.63)	14.24 (3.66)	17.98 (5.09)	-0.050 (-0.224, 0.098)	
Red Maple	24.97 (12.83)	25.05 (10.45)	25.74 (8.12)	14.13 (4.76)	12.73 (2.43)	23.04 (6.61)	0.038 (-0.091, 0.169)	
Other Trees	21.28 (8.77)	29.34 (11.95)	15.86 (5.51)	15.84 (6.24)	15.23 (3.52)	15.20 (4.65)	-0.016 (-0.201, 0.145)	
Smilax	2.56 (1.20)	5.05 (2.96)	5.48 (4.51)	0.32 (0.26)	0.00 (0.00)	1.71 (1.58)	0.060 (-0.077, 0.199)	
Grass	24.28 (11.45)	23.18 (11.64)	12.68 (6.93)	21.53 (8.22)	20.82 (8.42)	15.37 (4.51)	0.132 (-0.071, 0.310)	
Forb	15.10 (4.24)	21.83 (7.46)	18.21 (5.84)	8.64 (1.81)	12.01 (2.16)	23.21 (8.15)	0.131 (0.011, 0.251)	*
Total forage	96.76 (20.76)	114.30 (25.93)	88.13 (16.55)	79.04 (15.50)	75.02 (12.99)	96.93 (19.97)	0.050 (-0.067, 0.170)	
Proportion	0.18 (0.04)	0.16 (0.04)	0.18 (0.04)	0.12 (0.03)	0.12 (0.04)	0.17 (0.05)	0.056 (-0.074, 0.174)	
Blueberry	96.10 (23.92)	91.05 (22.10)	71.62 (16.26)	179.92 (28.18)	161.52 (26.04)	120.64 (21.30)	-0.043 (-0.216, 0.133)	
Other Shrubs	58.25 (38.90)	41.43 (30.84)	69.93 (47.26)	178.83 (87.51)	165.00 (71.08)	108.29 (55.51)	-0.089 (-0.374, 0.182)	
Teaberry	16.24 (8.36)	22.00 (11.36)	61.46 (30.45)	40.41 (13.51)	39.97 (14.69)	136.55 (49.10)	-0.047 (-0.303, 0.205)	
Bracken Fern	179.05 (49.98)	302.10 (102.70)	133.63 (46.75)	243.75 (40.54)	307.51 (57.65)	157.27 (35.48)	-0.060 (-0.267, 0.143)	
Other Fern	200.32 (45.00)	314.97 (69.84)	233.23 (56.75)	164.02 (45.64)	173.28 (46.65)	161.9 (47.74)	-0.028 (-0.350, 0.322)	
Non-forage	549.95 (92.65)	771.55 (158.96)	555.30 (116.31)	803.63 (107.14)	847.28 (120.82)	634.09 (73.85)	-0.024 (-0.115, 0.072)	

* Confidence interval excludes zero, indicating an effect of liming on that variable
