# Supplementary material for: Effects of Liming on Forage Availability and Nutrient Content in a Forest Impacted by Acid Rain
Source: PLoS One. 2012 Jun 28;7(6):e39755. doi: 10.1371/journal.pone.0039755 (PMC3386234; doi:10.1371/journal.pone.0039755)
Supplement: Table S2 — Percent dry weight (SE) of calcium (Ca), magnesium (Mg), potassium (K), phosphorus (P), and crude protein (CP) in the six categories of deer forage sampled in control and lime-treated sites before (2003) and after (2004, 2008) lime application, and the estimates of the time by treatment interaction term with confidence intervals (CI). (PDF) [file pone.0039755.s002.pdf]

Table S2. Percent dry weight (SE) of calcium (Ca), magnesium (Mg), potassium (K), phosphorus (P), and crude protein (CP) in the six categories of deer browse sampled in control and lime-treated sites before (2003) and after (2004, 2008) lime application, and the estimates of the time by treatment interaction term with confidence intervals (CI).

| | Control | | | Limed | | | | |
|---|---|---|---|---|---|---|---|---|
| | 2003 | 2004 | 2008 | 2003 | 2004 | 2008 | Interaction (CI) | |
| Oak | | | | | | | | |
| Ca | 0.631 (0.024) | 0.645 (0.037) | 0.598 (0.022) | 0.531 (0.035) | 0.548 (0.023) | 0.590 (0.0345) | 0.019 (-0.002, 0.039) | |
| Mg | 0.169 (0.013) | 0.170 (0.013) | 0.196 (0.009) | 0.175 (0.012) | 0.208 (0.019) | 0.342 (0.011) | 0.028 (0.019, 0.036) | * |
| K | 0.701 (0.020) | 0.756 (0.017) | 0.869 (0.021) | 0.736 (0.020) | 0.771 (0.029) | 0.822 (0.039) | -0.015 (-0.031, 0.003) | |
| P | 0.109 (0.007) | 0.139 (0.010) | 0.145 (0.005) | 0.117 (0.005) | 0.131 (0.004) | 0.133 (0.008) | -0.003 (-0.008, 0.002) | |
| CP | 11.16 (0.27) | 11.93 (0.17) | NA | 9.91 (0.19) | 11.17 (0.19) | NA | 0.499 (-0.230, 1.323) | |
| Red Maple | | | | | | | | |
| Ca | 0.663 (0.024) | 0.668 (0.023) | 0.622 (0.016) | 0.593 (0.023) | 0.626 (0.022) | 0.621 (0.018) | 0.012 (-0.003, 0.026) | |
| Mg | 0.133 (0.005) | 0.149 (0.005) | 0.171 (0.011) | 0.126 (0.005) | 0.210 (0.017) | 0.299 (0.007) | 0.024 (0.018, 0.032) | * |
| K | 0.622 (0.021) | 0.691 (0.035) | 0.717 (0.019) | 0.544 (0.023) | 0.637 (0.038) | 0.623 (0.024) | -0.005 (-0.024, 0.014) | |
| P | 0.121 (0.011) | 0.155 (0.011) | 0.139 (0.007) | 0.113 (0.007) | 0.161 (0.008) | 0.144 (0.006) | 0.002 (-0.005, 0.009) | |
| CP | 8.34 (0.17) | 9.69 (0.17) | NA | 7.30 (0.18) | 9.08 (0.13) | NA | 0.448 (-0.162, 1.099) | |
| Other Trees | | | | | | | | |
| Ca | 1.043 (0.158) | 0.920 (0.115) | 0.868 (0.048) | 0.758 (0.067) | 0.838 (0.058) | 0.880 (0.140) | 0.049 (-0.029, 0.135) | |
| Mg | 0.213 (0.003) | 0.195 (0.040) | 0.265 (0.005) | 0.213 (0.023) | 0.253 (0.063) | 0.408 (0.063) | 0.026 (0.003, 0.050) | * |
| K | 1.000 (0.020) | 0.915 (0.150) | 1.248(0.067) | 1.080 (0.060) | 1.055 (0.025) | 1.593 (0.523) | 0.053 (-0.113, 0.217) | |
| P | 0.155 (0.010) | 0.138 (0.012) | 0.140 (0.005) | 0.165 (0.015) | 0.160 (0.010) | 0.198 (0.017) | 0.009 (0.002, 0.017) | * |
| CP | 10.18 (0.66) | 10.58 (0.19) | NA | 10.23 (0.22) | 11.40 (0.11) | NA | 0.775 (-0.755, 2.230) | |
| Forb | | | | | | | | |
| Ca | 0.833 (0.036) | 0.671 (0.062) | 0.702 (0.048) | 0.648 (0.033) | 0.725 (0.037) | 0.729 (0.057) | 0.027 (-0.004, 0.061) | |
| Mg | 0.295 (0.022) | 0.270 (0.025) | 0.317 (0.023) | 0.296 (0.015) | 0.364 (0.033) | 0.479 (0.027) | 0.027 (0.012, 0.044) | * |
| K | 2.228 (0.133) | 2.376 (0.176) | 2.366 (0.116) | 1.916 (0.123) | 1.873 (0.152) | 1.662 (0.204) | -0.074 (-0.180, 0.028) | |
| P | 0.147 (0.012) | 0.173 (0.011) | 0.162 (0.009) | 0.154 (0.011) | 0.149 (0.004) | 0.161 (0.009) | 0.001 (-0.006, 0.008) | |
| CP | 11.76 (0.27) | 12.75 (0.32) | NA | 11.23 (0.30) | 11.46 (0.25) | NA | -0.753 (-1.821, 0.347) | |
| Grass | | | | | | | | |
| Ca | 0.233 (0.013) | 0.205 (0.005) | 0.155 (0.020) | 0.143 (0.012) | 0.150 (0.010) | 0.180 (0.010) | 0.022 (0.014, 0.034) | * |
| Mg | 0.128 (0.013) | 0.120 (0.005) | 0.088 (0.018) | 0.075 (0.005) | 0.103 (0.013) | 0.150 (0.010) | 0.022 (0.015, 0.030) | * |
| K | 1.763 (0.293) | 1.755 (0.215) | 1.918 (0.358) | 1.525 (0.055) | 1.478 (0.047) | 1.508 (0.253) | -0.034 (-0.236, 0.130) | |

| | | | | | | | | |
|---|---|---|---|---|---|---|---|---|
| P | 0.170 (0.030) | 0.153 (0.012) | 0.133(0.008) | 0.148 (0.028) | 0.150 (0.010) | 0.120 (0.015) | 0.001 (-0.012, 0.017) | |
| CP | 9.48 (0.23) | 10.45 (0.33) | NA | 8.53 (0.07) | 9.30 (0.16) | NA | -0.200 (-1.149, 0.635) | |
| Smilax | | | | | | | | |
| Ca | 0.583 (0.075) | 0.587 (0.063) | 0.321 (0.073) | 0.493 (0.063) | 0.541 (0.034) | 0.715 (0.00) | 0.100 (0.048, 0.142) | * |
| Mg | 0.136 (0.002) | 0.141 (0.006) | 0.071 (0.012) | 0.104 (0.024) | 0.144 (0.009) | 0.368 (0.00) | 0.065 (0.054, 0.079) | * |
| K | 1.069 (0.421) | 1.328 (0.117) | 1.198 (0.057) | 1.095 (0.185) | 1.678 (0.297) | 2.139 (0.00) | 0.182 (-0.054, 0.426) | |
| P | 0.093 (0.020) | 0.130 (0.009) | 0.077 (0.009) | 0.102 (0.012) | 0.150 (0.010) | 0.149 (0.00) | 0.014 (-0.006, 0.035) | |
| CP | 11.11 (0.42) | 12.35 (0.38) | NA | 8.87 (0.60) | 10.85 (0.50) | NA | 0.990 (-0.530, 2.701) | |

* Confidence interval excludes zero, indicating an effect of liming on that variable.
